# In silico analytico‐mathematical interpretation of biopolymeric assemblies: Quantification of energy surfaces and molecular attributes via atomistic simulations

**DOI:** 10.1002/btm2.10105

**Published:** 2018-09-26

**Authors:** Pradeep Kumar, Yahya E. Choonara, Viness Pillay

**Affiliations:** ^1^ Wits Advanced Drug Delivery Platform Research Unit, Department of Pharmacy and Pharmacology School of Therapeutic Sciences, Faculty of Health Sciences, University of the Witwatersrand Johannesburg South Africa

**Keywords:** biopolymers, drug delivery, molecular modeling, mucoadhesion, nanoformation, polyelectrolyte complexes, static lattice atomistic simulations

## Abstract

Static‐lattice atomistic simulations, in vacuum and solvent phase, have been recently employed to quantify the “in vitro—in vivo—in silico” performance‐correlation profile of various drug delivery systems and biomaterial scaffolds. The reactional profile of biopolymers was elucidated by exploring the spatial disposition of the molecular components with respect to the formulation conditions and the final release medium. This manuscript provides a brief overview of recently completed and published studies related to molecular tectonics of: (a) the nanoformation and solvation properties of the surfactant‐emulsified polymeric systems; (b) the formation and chemistry of polyelectrolyte complexes; (c) the effect of a plasticizer and/or drug on the physicomechanical properties of biomedical archetypes; (d) the molecular modeling templates to predict stimuli‐ and environmentally esponsive systems; and (e) the polymer‐mucopeptide complexes and intermacromolecular networks. Furthermore, this report provides a detailed account of the role of molecular mechanics energy relationships toward the interpretation and understanding of the mechanisms that control the formation, fabrication, selection, design, performance, complexation, interaction, stereospecificity, and preference of various biopolymeric systems for biomedical applications.

## 1 INTRODUCTION

The elucidation of mechanism(s) inherent to the performance of biopolymer‐based complex assemblies presents a significant challenge to pharmaceutical and biomedical scientists around the world with most of the studies *only* providing “qualitative estimation” based on “theoretical experience” and “quantitative experimental results.” The use of phrases such as “probably,” “may be,” “perhaps,” “theoretically,” “might be,” “supposed to,” and so on are frequently used to describe complex materials phenomena (such as crosslinking of polymers, drug release, or degradation) employing basic physicochemical, physicomechanical, and morphological analyses. Conversely, there are in silico *only* studies providing the theoretical knowledge of various material archetypes with only few being tested at the in vitro and fewer at the in vivo level. In addition, the computational methods applied in the advanced in silico studies are very expensive and extensive in terms of time and funding.^1^ Furthermore, there is a lack of considerable data and studies reflecting the in silico performance analysis of pharmaceutical systems post‐formulation and development. In a possible way to address the aforementioned challenges, a novel paradigm was developed for the in silico analytico‐mathematical interpretation of polymeric systems via quantification of energy surfaces and molecular attributes using atomistic simulations. Molecular mechanics simulations were employed for the “quantitative elucidation” of the mechanism inherent to polymeric archetypes. Sophisticated molecular modeling software was employed for 3D structure generation wherein the molecular structures were drawn in their syndiotactic stereochemistry and natural angle conformation. The overall steric energy was minimized through MM+, AMBER3, and MMFF94 force fields in conjugation with Polak–Ribiere conjugate gradient method (novel progressive convergence strategy). The results were presented as an analytico‐mathematical representation of potential energy surfaces with total energy composed of bond stretching, angle, and torsional contributions as bonding and van der Waals interactions, H‐bonding and electrostatic functions as nonbonding energies. The reactional profiles for component molecules and their complexes were elucidated by exploring the spatial disposition of the various component molecular attributes.^2^ This review report provides an outlook of various molecular modeling templates generated to date and provides a brief account of rationale and results obtained from these in silico studies. The details of the in silico methods employed in the studies are out of scope of this review and can be extracted from the individual references provided within the subsections. However, the definition and details of molecular mechanics energy relationship (MMER) model are provided in the following section.

### 1.1 Molecular mechanics‐assisted model building and energy refinements

The MMER model for the energy factor in various molecular complexes can be written as:

(1) $$E_{\text{molecule}/\text{complex}}=V_{\sum }=V_{b}+V_{\theta }+V_{\phi }+V_{\mathrm{ij}}+V_{\mathrm{hb}}+V_{\mathrm{el}}$$

where *V*_∑_ is related to total steric energy for an optimized structure, *V*_b_ corresponds to bond stretching contributions (reference values were assigned to all of a structure's bond lengths), *V*_θ_ denotes the bond angle contributions (reference values were assigned to all of a structure's bond angles), *V*_ϕ_ represents the torsional contribution arising from deviations from optimum dihedral angles, *V*_ij_ incorporates van der Waals interactions due to nonbonded interatomic distances, *V*_hb_ symbolizes hydrogen‐bond energy function, and *V*_el_ stands for electrostatic energy.

In addition, the total potential energy deviation, ΔE_Total_, was calculated as the difference between the total potential energy of the complex system (A/B) and the sum of the potential energies of isolated individual molecules (A, B), as follows:

(2) $${\mathrm{\Delta E}}_{\text{Total}\left(A/B\right)}={E_{\text{Total}}}_{\left(A/B\right)}–\left[E_{\text{Total}\left(A\right)}+{{E_{\text{Total}}}_{(B}}_{)}\right]$$

## 2 NANOFORMATION AND SOLVATION PROPERTIES OF POLYMERIC NANOSYSTEMS

**Figure 1:**
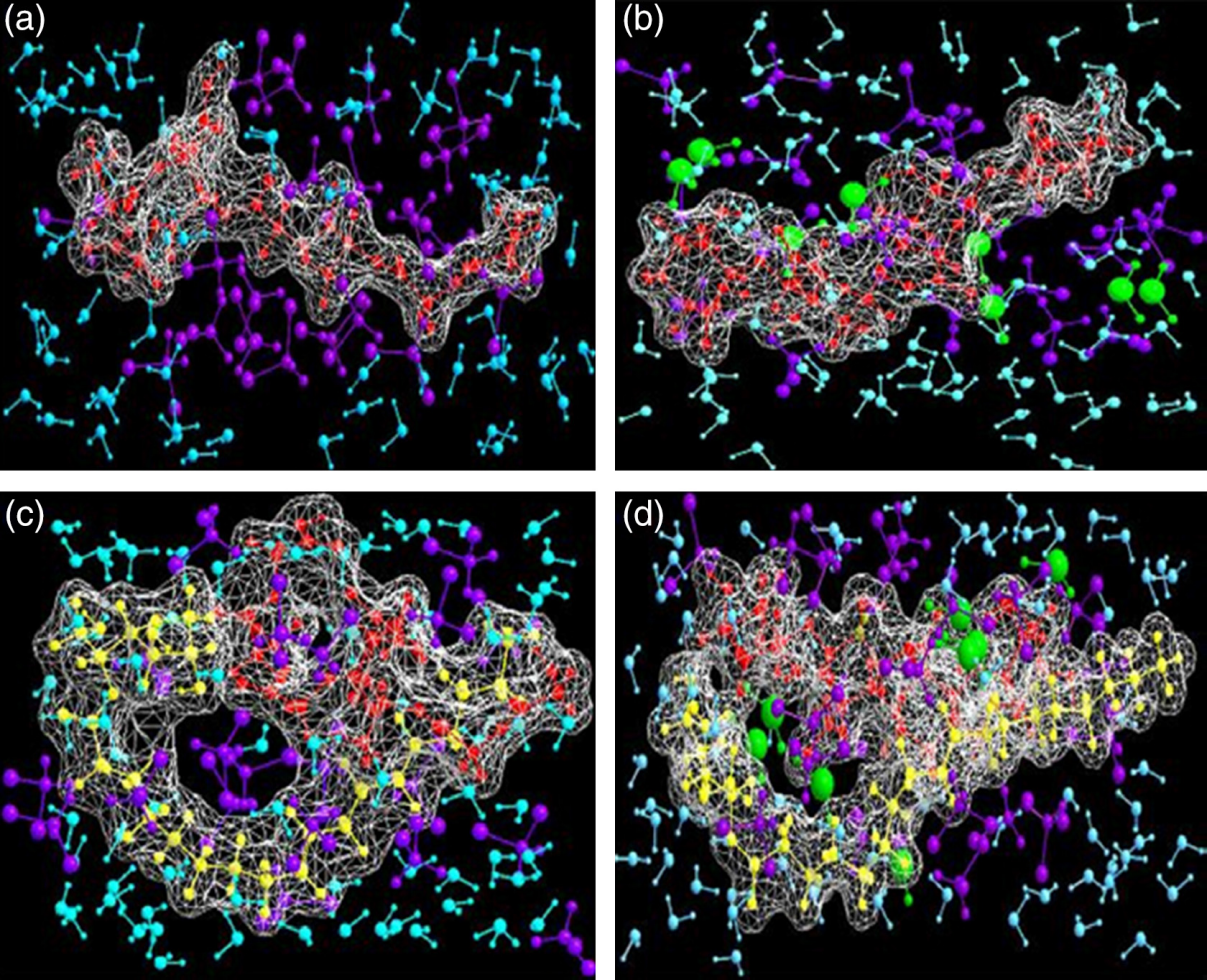
Connolly molecular electrostatic potential surfaces in wire mesh display mode showcasing the four nanoparticlulate systems: (a) ESE; (b) ESSE; (c) RECG; and (d) RESCG. Color codes: PLGA and alginate (red), surfactant (yellow), DCM molecules (violet), water molecules (cyan), and Ca^2+^‐ions (green) (Reference 5; reproduced with permission from Elsevier B.V. Ltd. © 2011)

The formation and formulation of nano‐based systems can be achieved either through relatively simple self‐assembly (nanoliposomes or nanopeptides) or via complex emulsification‐homogenization (polymeric nanoparticles). In either case, the stabilization of the nanosystem so formed is of utmost importance and can be achieved in several ways such as by using a surfactant, a polymeric stabilizer (such as polyvinyl alcohol [PVA]) or even via crosslinking‐co‐rigidification of the polymeric platform.^3^, ^4^ Choonara and coworkers reported poly(lactic‐co‐glycolic acid) (PLGA) nanoparticles formulated using two different approaches based on whether a surfactant was used to stabilize the colloidal phase: (a) emulsion‐solvent‐*surfactant*‐evaporation (ESSE) and 2) emulsion‐solvent‐evaporation (ESE). The MMER report of these complex colloidal phases concluded that the presence of a surfactant such as sorbitan monooleate can provide the much needed conditions for the formation of uniform polymeric nanoparticles. In the ESSE paradigm, the presence of surfactant led to an increase in thermodynamic energy at the solvent/particle interface and hence the coagulation and aggregation of the nanosystem was prevented. In addition, the electrostatic and steric repulsions in case of ESSE were in the range of 1,000 and 200 kca/mol, respectively, under solvent phase simulations. Similarly, alginate nanoparticles were prepared using rigidification–gelification approach with or without adding a surfactant: (a) reverse‐emulsion‐cationic‐gelification (RECG) and (b) reverse‐emulsion‐*surfactant*‐cationic‐gelification (RESCG). In addition to the thermodynamic shielding and repulsions discussed above, the presence of surfactant in RESCG provided a hydrated palisade layer structure which played a major role in crosslinking induced stabilization of the nanosystem termed as—“crosslinking stabilized–stabilized emulsion.” The presence of this hydrated layer increased both the polarizability and refractivity coefficient of the RESCG matrices leading to the formation of a rigidified structure with least probability of colloidal aggregation or flocculation (Figure 1).^5^ The modeling results provided a potential relationship between a change in the in vitro zeta potential and reduction of particle size on addition of a surfactant to the polymeric nanosystem. The proposed in silico electrostatic and steric profiling in a solvated system may act as a template for future prenanoformulation design. du Toit and coworkers predicted the nanoformation and cellular internalization profiles of the lipoidal–chitosan–poly(ε‐caprolactone) nanosystem using MMER. The geometrical optimization confirmed the formation of a helical bimolecular structure due to extensive nonbonding hydrophobic interactions within the CHT‐PCL matrix. The addition of a lipid phase (phospholipids) such as disteroylphosphatidylcholine (DSPC) and/or distearoylphosphatidylethanolamine (DSPE) provided the much needed hydrophilic–lipophilic balance required for optimal performance of the nanosystem. Interestingly, the addition of DSPC to the CHT‐PCL matrix rendered it more lipophilic while DSPE provided a relatively hydrophilic character. The addition of phospholipids to the polymers increased their aqueous‐interaction capability due to increased electrostatic interactions. Therefore when both DSPC and DSPE were added together to the nanomatrix, the final closed‐and‐defined network structure was represented as an amphiphilic lipoidal aggregate with the required “hydrophilic–lipophilic balance with lipophilicity on the higher side” for high cellular internalization.^6^ The solvated MMER simulation provided an important aspect of assessing the hydrophilic–lipophilic‐balance within a lipo‐polymeric matrix wherein the energy destabilization data (Chit–PCL–DSPC–H_2_O > Chit–PCL–DSPC–DSPE–H_2_O > Chit–PCL–DSPE–H_2_O) directly corroborated with the ex vivo internalization findings.

On similar grounds, DSPE‐PEG/DSPC‐based nanoliposomes with cholesterol (CHOL) as the lipidic component were reported earlier. The presence of methoxy‐PEG moiety led to the formation of H‐bonding within the phospholipid complex. However, this still was not sufficient to form a thermodynamically stable nanosystem. As determined by the 3D geometrical conformation and stabilization via molecular modeling, the addition of CHOL provided the much needed space filling properties to the ternary system wherein the van der Waals space of the molecular complex contributed to matrix stabilization. This change in H‐bonding dominated system to hydrophobically stabilized system was further responsible for the self‐assembly of the complex into nanoliposomes (nanoformation) when hydrated in phosphate buffered saline.^7^ Furthering the modeling paradigm of the effect of PEG on nanoformation, Tomar and coworkers discussed how using an amphiphilic PEG can assist in nanostabilization and nanoperformance. Poly(lactic acid) (PLA) was grafted with different molecular weight PEG molecules (PEG_575_, PEG_2000_, and PEG_4000_) and were then loaded with insulin as the model bioactive. As explained above, PEG provided the much needed hydrophilicity to the lipophillic PLA matrix and hence nanoformation within the pluronic‐based aqueous phase. Similar to CHOL, the PEG molecules played the space‐filling role and hence led to the stabilization of nonbonding hydrophobic interactions leading the efficient encapsulation of insulin in the nanomatrix. Interestingly, when MMER was applied to study the release of insulin from the nanomatrix, the presence of aqueous phase (with no cosolvent or surfactant present) hydrated the PEG component leading the entry of water into the nanomatrix and hence disturbing the hydrophobic forces thereby releasing the encapsulated bioactive. However, this concept failed in case of low molecular weight PEG‐diacrylate where the drug release was least on weight basis. The MMER conducted for all three variants in aqueous medium concluded that the insulin showed molecular interactions with the functional groups (–COOH and –NH_2_) present in the polymer matrix. This further confirmed that insulin released from PLA‐PEG_575_ matrix will be slowest as low molecular weight PEGDA will have more NH_2_ functional end groups than the higher molecular weight ones.^8^ In conclusion, the in vacuo and in solvo MMER analyses for nanoformulations provided an important insight into the hydrophilic–lipophilic character of the nano‐self‐assembly (via energetic contributions) as well as highlighted the important of adding space filling additives to the stabilization such complex systems. However, effective selection of the molecular weight and chain length of various incompatible systems (such as cholesterol/lipids vs. polymers) needs to be optimized further to obtain better geometrical optimization and conformations.

## 3 MOLECULAR INTERACTIONS INHERENT TO POLYELECTROLYTE COMPLEXES

Polyelectrolyte complexes, as the name suggests, are complex molecular architectures composed of oppositely charged molecules. As the charged functional groups are “consumed” in the process, the inherent solubility and functionality of the individual molecules are reduced and hence an insoluble but hydrated and swellable matrix is formed.^9^ In silico modeling of these complex systems has provided a unique insight into their performance mechanism for biomedical applications. In a series of studies, Ngwuluka and coworkers provided the first‐ever step‐by‐step formation and synthesis of a polyelectrolyte complex. The research entailed an interpolyelectrolyte complex between anionic sodium carboxymethylcellulose (NaCMC) and a cationic polymethacrylate polymer (Eudragit E100). To decode the synthesis of the complex, a novel technique, namely, Intermittent Snapshot Modeling Approach was introduced. A snapshot was taken at the end of a major conformational change and the relevant energy values were recorded followed by further minimization of geometrical energy. This way the authors managed to deduce the polyelectrolyte complexation into four different stages as follows: (a) initial stage: the presence of intramolecular bonding within NaCMC and the absence of any intermolecular interactions with Eudragit E100; (b) intermediate stage: first evidence of formation of intermolecular bonds with a relative decrease in NaCMC intermolecular interactions; (c) breaking point: formation of complex structure with thickening of the reaction medium due to network entanglement; and (d) final product: a homogenous polyelectrolyte complex formation with a perfect balance of intramolecular and intermolecular interactions. The in silico results so obtained were well corroborated with the in vitro rheological analyses and the stages were assigned at 30 s, 1 hr, stopping of magnetic bar movement, and final homogenous product.^10^, ^11^, ^12^ The formation of NaCMC‐Eudragit E100 I.E. was accompanied by dense H‐bonding and van der Waals interactions. This was further confirmed by lower refractivity, reduced surface‐to‐volume ratio, and high‐density of 0.442 amu/Å^3^. In addition, the molecular dynamics simulation supported the energy stabilization and it was concluded that “the potential energy decreased with an increase in the kinetic energy, obeying the well‐known behavior of high underdamping harmonic oscillator.”^12^

**Figure 2:**
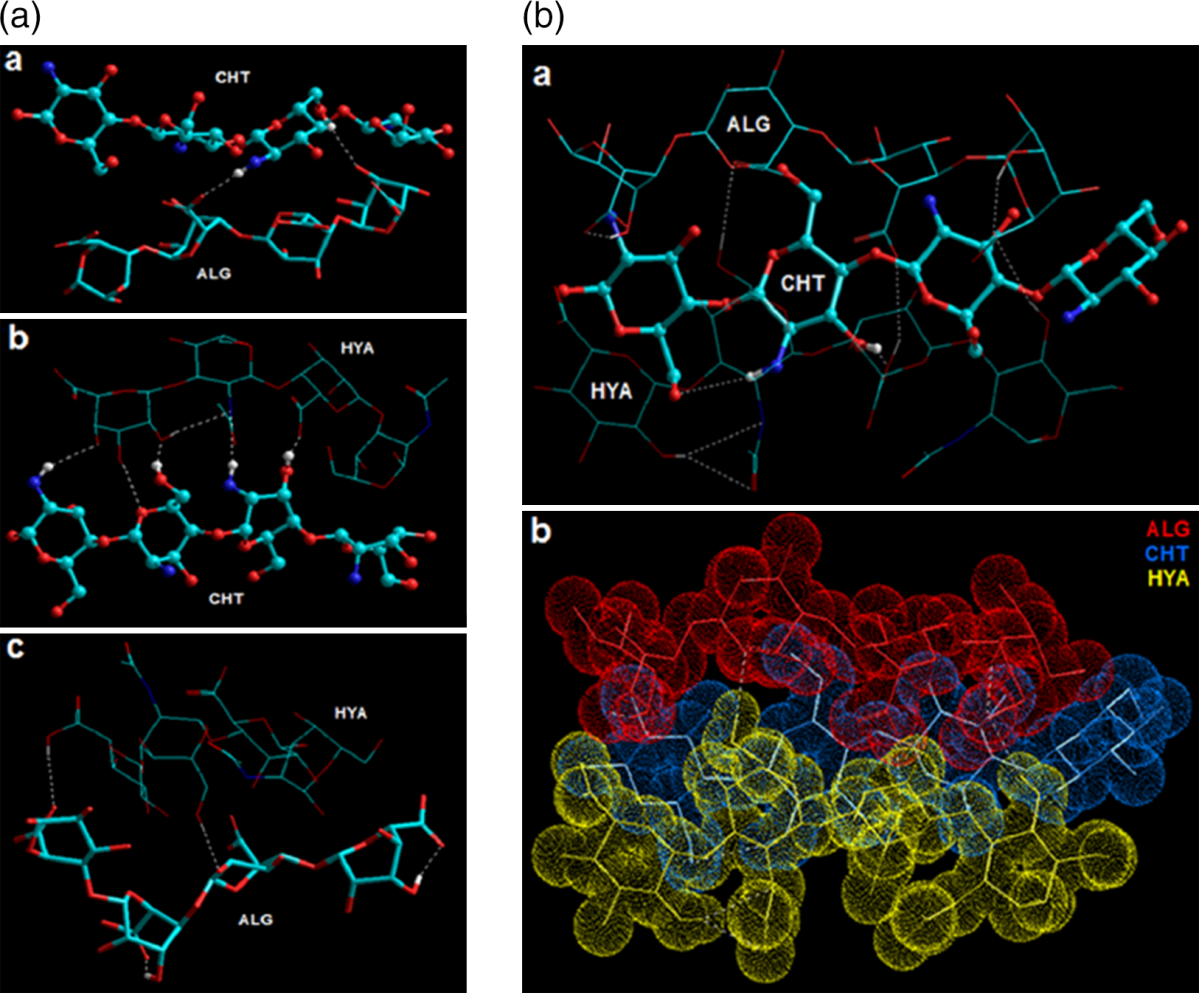
(a) Energy minimized geometrically conformation displaying the bipolymeric complex of (a) alginate (tubes) and chitosan (ball‐and‐tube); (b) chitosan (ball‐and‐tube) and hyaluronic acid (stick); and (c) hyaluronic acid (stick) and alginate (tubes) after molecular mechanics simulations. Color codes for elements: carbon (cyan), hydrogen (white), nitrogen (blue), and oxygen (red). (b) Energy minimized geometrically conformation displaying the tripolymeric complex of alginate (tubes), chitosan (ball‐and‐tube), and hyaluronic acid (stick) after molecular mechanics simulations. Color codes for elements in (a): carbon (cyan), hydrogen (white), nitrogen (blue), and oxygen (red). Color codes for structures in (b): alginate (red), chitosan (blue), and hyaluronic acid (yellow) (Reference 14; reproduced with permission from springer nature © 2015)

Bawa and coworkers reported a molecular model of a tripolymeric ionic quadrilateral (TPIQ) wherein chitosan and pectin formed the cationic and anionic counterions, respectively. The third polymer employed was a partially hydrolyzed polyacrylamide (HPAAm) which acted as an anionic molecule as well as a cationic molecule in close vicinity of chitosan (CHT) and pectin (PEC), respectively. The molecular modeling simulations presented a van der Waals space overlap and the hydrogen bonding between the interaction molecules confirmed that –NH^3+^ (CHT and HPAAm) and –COO^−^ (PEC and HPAAm) functional groups formed the TPIQ network via structure‐selective binding of saccharidic moieties. The in silico results concluded that the geometrical adjustment of polymeric chains to form the ionic complex is due to the minimization of Coulombic attractions and van der Waals forces. This further confirmed the in vitro controlled release of a highly soluble drug (diphenhydramine) due to matrix curing and formation of a “deforming‐type” matrix.^13^ Recently, Bijukumar and coworkers modeled a multimolecular alginate:chitosan:hyaluronic acid (ALG:CHT:HYA) polyelectrolyte complex. The MMER analysis provided an excellent insight into the composition of the nanostructured system wherein the alginate chains carried the drug molecule, hyaluronic acid provided proposed inflammation sensitive coating over the carrier molecule, and finally chitosan acted as the bridging agent to congeal the anionic molecules together—forming a stable and functional TPIQ architecture. Interestingly, HYA (being amphi‐ionic) provided the much needed ionic balance within the TPIQ and hence the formation of a well‐connected 3D architecture supported by extensive H‐bonding among the bimolecular (ALG:CHT, CHT‐HYA, and HYA:ALG) and trimolecular (ALG‐CHT‐HYA) regions and involved –COOH/‐COOH, –OH/–OH, –NH/–OH, –OCO–OH, –COOH–OH, and –COOH/–NH functional groups (Figure 2).^14^ The above discussion clearly confirms the important role played by amphi‐ionic or amphoteric systems in generation of effective polyelectrolyte complexes for biomedical applications.

In a very important study, a mucopeptide was employed as an excipient to form a bimolecular complex with pectin. Although there were several earlier reports discussing the interactions of pectin with mucin, the molecular interactions inherent to this complex were not yet known or elucidated. The researchers via MMER established that there might be a possibility of formation of a blend‐co‐complex within this proteo‐saccharide matrix. Although the –COOH functionalities of the molecules lead to steric repulsion and a strained network, this initiated the much needed “neighboring” of the counterions: –NH^3+^ of mucin and –COO^−^ of pectin chains – overcoming the torsional barriers required for a stable network. It was further established that this interaction is concentration dependent and saturation of functional groups can be achieved by carefully manipulating the biomaterials' quantities.^15^ The above discussion confirms the importance and applicability of MMER in determining the complex interactions within an interpolymeric system consisting of oppositely charged polymers and components. However, there is a minor limitation of this energy relationship paradigm with respect to its applicability in zwitterionic systems as well as differentiating between intermolecular and intramolecular energy components which needs to be further explored.

## 4 EFFECT OF PLASTICIZERS ON PERFORMANCE OF BIOMEDICAL ARCHETYPES

**Figure 3:**
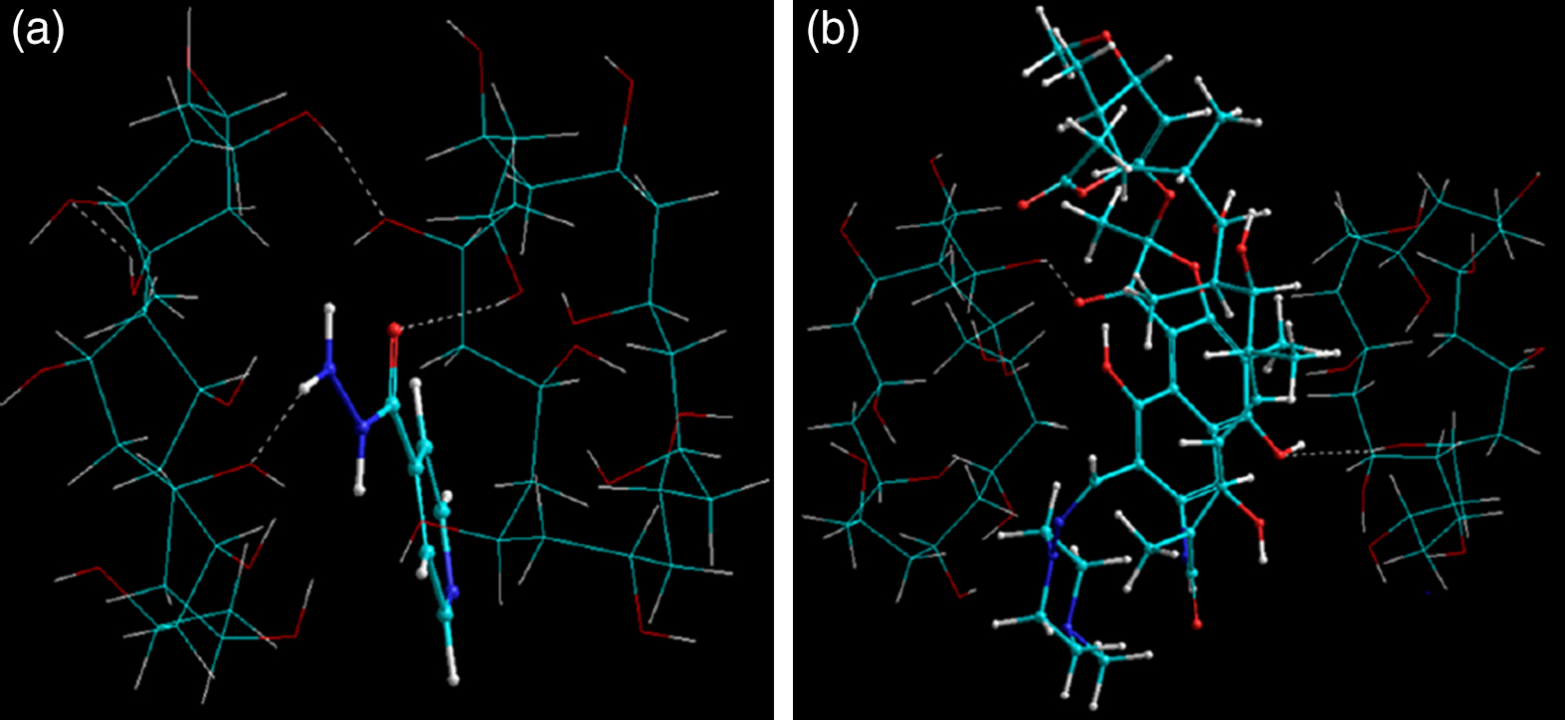
Visualization of energy minimized geometrical preferences of two PVA molecules in conjugation with (a) an isoniazid molecule and (b) a rifampicin molecule showcasing the intramolecular and intermolecular interactions after molecular simulations in vacuum. Color codes for elements: C (cyan), O (red), N (blue), and H (white) (Reference 17; Reproduced with permission from IOP Science Publishers © 2012)

Polymeric fibers provide distinctive advantages in terms of size selection and mechanical properties which further define the final biomedical application. For example, nanofibrous mats are widely employed for wound dressing and oral drug delivery, microfibers for sutures, and macrofibers for periodontal diseases. For these fibers to perform optimally mechanically, addition of plasticizers is a common practice and follows the same principles as for the plasticization of polymeric films. However, given the large surface area and fine diameter size of the fibrous systems, the addition of any additives needs to be carefully controlled. For example, in case of electrospun nanofibrous systems, the electrospinning process involves careful selection of polymers and additives which in in turn affect the processing variables. As the final size of the fibers is in the range of nanometers with high surface area, addition of even a small quantity of drug may lead to reassignment of processing variables.^16^ Shaikh and coworkers prepared PVA nanofibrous mats using electrospinning as the fabrication technique. The nanofibers were loaded with drugs (rifampicin [RIF] and isoniazid [INH]) by dispersing the drugs in electrospinning solution following by postfabrication crosslinking of the fibers using glutaraldehyde vapors. Interestingly, it was noticed in vitro that the drugs acted as nontraditional plasticizers and significantly affected the physicomechanical‐tensile properties of the nanofibers (Figure 3). MMER was used to explore this interesting finding with a directed reference to available molar volume (MV) between the polymer chains and the related cohesive energy density (CED). It was deduced that CED is inversely proportional to MV and is important for the elasticity and rigidity of the nanofibers: higher the CED, lower the elasticity and vice versa. Addition of drugs to the nanofibrous matrix reduced the van der Waals interactions to a significant amount and hence decreased the CED allowing the polymer chains to slide over each other. This lead to a reduction in Young's modulus and was further dependent in the nature of the drug. For INH, a water soluble drug, the plasticizing effects were very dominant as the drug easily penetrated molecularly within the polymer matrix. However, RIF on the contrary demonstrated not so prominent effect on the nanotensile properties and even increased Young's modulus in line with drug free fibers. The reasons for this were proposed to be decrease in porosity in the nanofibers, filling of cracks, increase in cohesion and even molecular complexation.^17^ Dott and coworkers also developed PVA‐based electrospun nanofibers with hydroxypropylmethyl cellulose (HPMC) as the aqueous soluble‐swellable polymer and glycerin (GLY) as the plasticizer. The initial molecular models were developed “employing a derivative approach based on average‐density function of the pure systems.” The geometrical conformation of this trimolecular complex demonstrated that GLY was positioned into and onto the PVA‐HPMC bipolymeric network forming an energetically stable, plasticized GLY‐HPMC‐GLY‐PVA‐GLY structure. The MMER analysis in this case strengthened the notion of rigorous geometrical adjustments within the polymeric networks to accommodate a plasticizer which in return allows for the relatively free movement between the polymeric chains and layers.^18^

As opposed to nanofibrous mats, micro‐ and macro‐fibers are fabricated using the extrusion‐gelification technique. The macrofibers in particular are used individually and afford different set of challenges such as holding the fiber using a tweezer (e.g., for suturing) or simply implanting around a tooth for localized periodontal drug delivery. Johnston and coworkers, described crosslinked‐co‐plasticized, drug‐loaded, alginate fibers for localized periodontal drug delivery.^19^, ^20^ As described above, the addition of individual drug, ciprofloxacin or diclofenac, decreased Young's modulus of the prepared fibers owing to the now known reduction in CED. Interestingly, the nanotensile properties were regained when both the drugs were added together to the fibers. According to the MMER generated, this was attributed to: (a) increase in solubility parameter (δ) due to achievement of saturation solubility and hence a proportional increase in CED (CED ∝ δ^2^),^21^ (b) occlusion of pores accompanied by decreased porosity and hence increased Young's modulus according to Spriggs' equation, and (c) increased crystallinity of the fibers leading to intermolecular interactions and rigidity.^19^ The addition of conventional plasticizer such as glycerol was accompanied by stabilization of the polysaccharide matrix both kinetically and thermodynamically with a negative energy of mixing and formation of close range (2.2052–3.0464 A°) intramolecular and intermolecular H‐bonds.^20^

Furthering the modeling of plasticizers within polymer matrices, Cooppan and coworkers explained the importance of a solid plasticizer's concentration on the optimal performance of a Eudragit E100‐based memblet system. The MMER obtained from solvent system simulation confirmed that addition of PEG4000 at a very high concentration (60% wt/vol) may lead to formation of charged regions with less solvent accessibility. These charged regions may further distort the electrostatic balance of the polymeric matrix causing repulsion and a strained network. However at 30% wt/vol PEG4000 concentration, the PEG‐enclatherated polymethacrylate system was comparatively more geometrically stabilized with defined viscoelastic regions and consistent drug release profile.^22^ In a unique “co‐blending‐co‐plasticizing” strategy, Jones and coworkers used HPMC and Eudragit RS100 to make a bipolymeric buccal film and added two plasticizers, namely, glycerin (GLY) and triethyl citrate (TEC) to plasticize the individual polymers, respectively. This complex system demonstrated varied drug release profiles dependent on the concentration of the components. An increase in HPMC/GLY showed an increase in drug release while a prolonged drug release was observed with an increase in EUD/TEC component. The conformational profile of the buccal film matrix confirmed that TEC is better accommodated into the molecular space of EUD (as compared to that of GLY within HPMC) reducing solvent accessibility and increasing energetic stabilization. Such stabilized structure is less prone to diffusional or erosional release of the drug molecules.^23^ Although the MMER analysis in case of plasticized polymeric systems provided an important insight into the CED between the polymer chains; the applicability of the modeling paradigm to simulate the liquid–solid interface within the plasticized system warrants further investigation and revised algorithms.

## 5 ENVIRONMENTALLY AND INHERENTLY RESPONSIVE DRUG DELIVERY SYSTEMS

**Figure 4:**
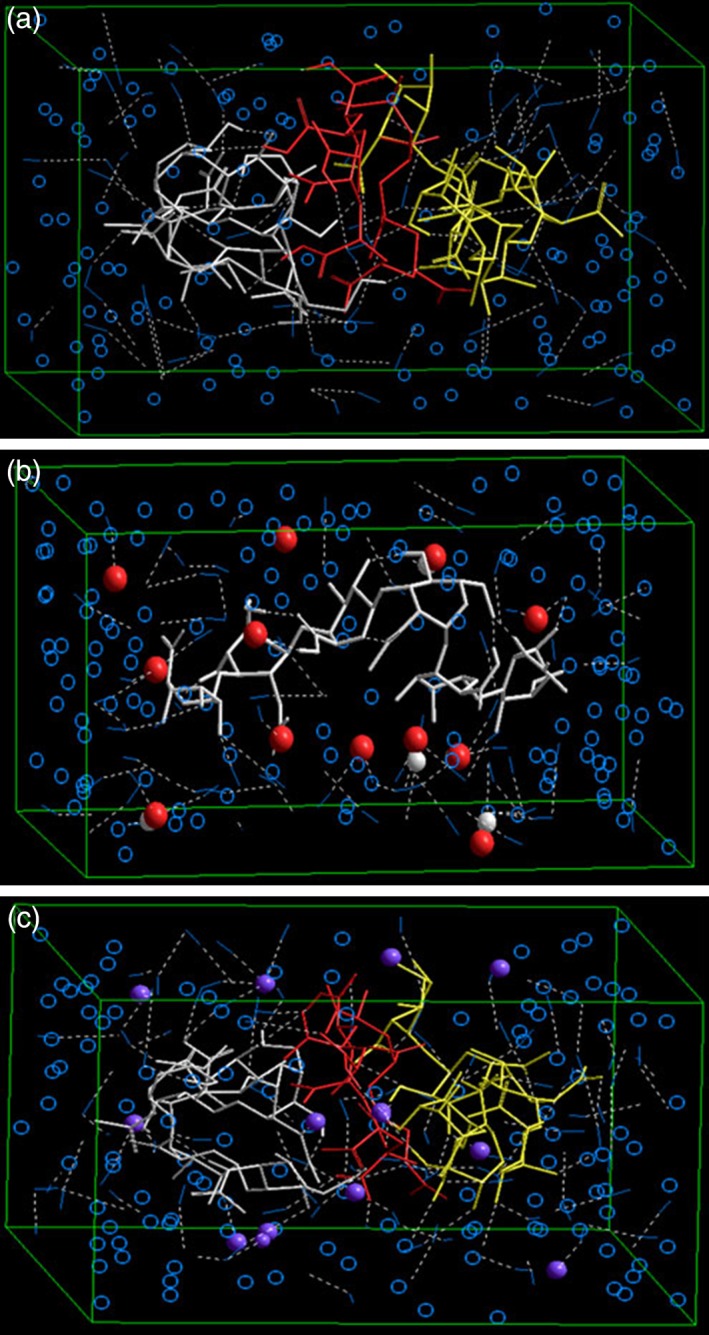
(a) Visualization of the geometrical preference of the tripolymeric complex consisting of alginate (yellow), polyacrylic acid (red), and hyaluronic acid (white) after molecular simulation in a solvated system consisting of water molecules (blue molecules). (b) Visualization of geometrical preference of hyaluronic acid (white tubes) and hydroxyl ions (red balls). (c) Visualization of geometrical preference of tripolymeric complex consisting of alginate (yellow), polyacrylic acid (red), and hyaluronic acid (white) in response to addition of hydroxyl ions (purple balls) after molecular simulation in a solvated system consisting of water molecules (blue molecules) (Reference 29; reproduced with permission from springer nature © 2013)

Stimuli and environmentally responsive drug delivery systems form an important aspect of current drug delivery strategies given the “delivered only when needed” characteristic of these specialized platforms. These systems can be easily, but nonconclusively, classified as thermo‐, pH‐, electro‐, light‐, ion‐, oxidation‐, and enzyme responsive.^24^ The authors have thus far provided interesting evidence of matrix responsiveness via MMER for three different stimuli as well as the behavior of drug delivery system in response to the biological clock as described below:

1. Enzyme responsive drug delivery: Bawa and coworkers described a stimuli‐synchronized matrix for localized colonic delivery of an anti‐inflammatory drug. The matrix systems were composed of tripolyphosphate crosslinked chitosan dispersed within a BaSO_4_‐stabilized pectin matrix. The matrix provided localized delivery if mesalamine in the colon environment and under the influence of colonic enzymes polygalacturonase and β‐glucosidase. The colonic enzyme fragments employed in the study were generated via deduction of information corresponding to the active catalytic amino acid sequences for human acid β‐glucosidase and pectin Lyase C. The MMER data suggested that the enzymatic sequences interacted with the functional moieties of the polysaccharide molecules and relaxed the close interatomic contacts leading to degradation of the tablet matrix and hence release on the drug in the colonic environment.^25^
2. Electroresponsive drug delivery: Indermun and coworkers developed a bipolymeric interfacially plasticized electroresponsive hydrogel composed of a polyacrylic acid‐poly(vinyl alcohol) (PAA‐PVA) semiinterpenetrating polymer network impregnated with poly(ethyleneimine) and 1‐vinylimidazole as electroactive moieties. The molecular mechanics simulations were carried out under solvent conditions and electrostatic charges were applied three‐dimensionally to the matrix system. The researchers theorized that polymers undergo a very complex electro‐induced organization and then re‐organize when electrical stimulation was removed. The jump‐diffusional behavior shown by the electroactive molecules played a major role in drug release on the application of electric field (see Table 1 for the steps).^26^ Earlier in 2011, Tsai and co‐workers modeled bipolymeric (PVA‐polyaniline) electroresponsive under solvent phase and directional electric fields were applied at external electric fields of 0.01–0.05 a.u. The variation in total steric energy was directly proportional to increase in applied electric field and it was concluded that the applied field led to bond stretching close to the point of dissociation causing the ON–OFF drug release profile reported in in vitro studies. Furthermore, the bipolymeric matrix “hopped” between helical and coiled structures during the ON–OFF electrostimulation.^27^ More recently, the electro‐stimulability of a PVA, PEG, and polystyrene sulphonate (PSS) complex was tested for application as an injectable electro‐conductive implant. The molecular model revealed that the three molecules formed a sandwich‐type ethylene–vinyl–sulfonate globular complex with PSS in between PEG and PVA layers. The application of electric field disturbed the strong nondecomposing, non‐unwinding, or non‐eroding complex to a loose adduct causing a pulsatile “ON–OFF” drug release.^28^
3. Inflammation induced drug delivery: The biochemical processes inherent to inflammatory conditions involve the generation of free radicals at and around the injured or diseased site. These free radicals are capable of interacting with functional groups of certain biopolymers and can be employed for redox‐responsive drug delivery. du Toit and coworkers designed an intelligent intraocular implant with an in‐built mechanism of polymeric erosion in close vicinity of free‐radicals resulting in environmentally sensitive drug release. For molecular modeling analysis, free radicals were introduced as hydroxyl ions into the solvated phase (149 water molecules). Hyaluronan (HYA) polymer chains in particular showed geometrical variations and interactions with the hydroxyl ions (Figure 4). The tripolymeric complex consisting of alginate, PAA and HYA polymer chains also responded to hydroxyl ions in conjugation and were attributed to the release of the bioactive under inflammatory conditions in vivo.^29^
4. Chronotherapeutic drug delivery: The basic concept of achieving a successful chronotherapeutic system is the perfect selection and combination of polymers and excipients with varied (or delayed) dissolution and/or degradation profiles and the arrangement of these selected entities into a layered or multicomponent platform.^30^ Khan and coworkers designed a multilayered, multidisc, oral tablet to potentially achieve a “drug delivery system with desired release profile (DDSDRP).” The molecular modeling template generated for the study provided important insights as to how the polymers should be combined to achieve DDSDRP. Among the possible combinations, it was established that higher energy of binding in ethyl cellulose‐hydroxy ethyl cellulose (EC‐HEC) layer as compared to pectin‐microcrystalline cellulose (PEC‐MCC) layer led to a delayed release of the drug from EC‐HEC. This means that PEC‐MCC combination can form the outer layer and be loaded with the immediately needed drug while EC‐HEC can be encapsulated into the core of PEC‐MCC for a drug required to be released later. This way the in silico designing can assist in effective preformulation of DDSDRP.^31^

**Table 1:** Electro‐influenced geometrical organization‐reorganization theory for bipolymeric interfacially plasticized electroresponsive hydrogel (Reference 26; reproduced with permission from Elsevier B.V. Ltd. © 2014)

The organization	The Reorganization
Polymeric chains organize with respect to the direction and strength of electric field: electric field application → polymer chains organization → increase in static energy due to electron transfer reaction → molecular alignment → planar structural conformation → reduced networking → electroresponsive drug release	Polymeric chains reorganize with respect to surrounding polymer molecules/plasticizer/solvent molecules via “LOCs”: Intrinsic interactions → local oriental correlations → change in reaction co‐ordinates → solvent relaxation → polymer chains reorganization → decrease in static energy values → increased networking → drug retention
VI molecules tend to drift close to the hydrogen‐bonding sites The molecules display a critical “jump diffusional behavior” The polymer chains vibrate within a microenvironment and then move to new micromolecular sites The jump motions were concentrated along varied locations in the vicinity of electrostatic charged spots attracting the water molecules	The molecular complex does not show the fluctuation flexibility The molecular components demonstrate a differential spatial variation leading to geometrically optimized and energetically minimized structures via two principle component interactions, one among the polymer/plasticizer molecules and the other among the complex and solvent molecules leading to a well‐organized and highly stable molecular architecture

Abbreviation: LOCs = local oriental correlations.

Molecular modeling studies of such complex systems are very limited in the literature. The major limitation of achieving the “responsiveness” in silico stems from the unavailability of modeling platforms for perfect environmental simulation and control and need innovative solutions to elucidate the responsive behavior.

## 6 THE PROTEIN‐POLYSACCHARIDE AND POLYMER‐MUCOPEPTIDE COMPLEXES

This section details a series of mucoadhesion and interaction molecular modeling studies carried out by the authors over the years (2010 onward; Table 2). Mucoadhesion is a very important aspect of oral drug delivery (in particular peptide delivery) when the drug release and absorption are targeted for the small intestine. Given the close proximity to the intestinal membrane, the mucoadhesive devices are developed to provide a high concentration gradient at the site of absorption thereby enhancing absorption as well as protection from the enzymatic degradation of the bioactives.^37^ Although there are abundant studies and data describing the “probable” muco‐interacting functionalities within a biopolymer or biopolymeric matrices; the confirmatory visualizations of these interactions are lacking. For the studies below, the authors employed a glycoprotein sequence homologous to mucous extracellular matrix. The mucopeptide so developed was energetically and geometrically minimized to give a globular protein structure mimicking the native mucous network.^38^

**Table 2:** The in silico mucopeptide‐biomolecular interaction profile of biopolymers

Biopolymer(s)	Biomolecular interactions	Device description	Reference
Polyvinyl alcohol Poly(acrylic acid)	Van der Waals forces and H‐bonding; Hydrophobic interactions of the –CH_3_ groups of MUC with residues of oxygen atoms of the polymers	Intravaginal bioadhesive polymeric device	32
Alginate Pectin Poly(acrylic acid)	Stress transduction for energy minimization Van der Waals forces, H‐bonding and electrostatic interactions Ca^++^ crosslinking destabilized the interactions and decreased the mucoadhesion potential	Dual mechanism gastrofloatable and gastroadhesive delivery system	33
Poly(vinyl alcohol) Hydroxypropyl methylcellulose	Van der Waals forces, H‐bonding and electrostatic interactions Mucoadhesion due to the biopolymers was accompanied by a “region of maximum”—dependent on the concentration of the polymer	Mucoadhesive electrospun nanofibrous matrix for oramucosal drug delivery	18
Hydroxypropyl methylcellulose Eudragit RS100	–OH groups of HPMC to the –COOH and –NH_2_ groups of MUC Quaternary ammonium groups of EUD seemed to form the much needed electrostatic interactions to impart mucoadhesivity An increase in HPMC concentration may lead to a decrease in mucoadhesion	Monolayered multipolymeric buccal films	23
Pectin	Rotation of PEC and MUC residues creating strain due to steric interactions Inclusion of bond length and angle adjustment Steric interactions caused functional groups of PEC (–OH and –COOH) and MUC (–OH, –COOH, and –NH_2_) to interact Decrease in the stabilization energy values with each subsequent addition of a PEC macromolecule	Interpenetrating proteo‐saccharide hydrogel network	15
Pullulan	–CO…NH2– and –CO…OH– interactions between PLLN and mucopeptide, respectively a large fraction of the surface required to establish connectivity between chemically transformed regions Strong H bonding in PLLN‐MUC with a bond lengths of <2 Å	Interpolyelectrolyte gastroretentive matrix	34
Chitosan Poly(acrylic acid) Gelatin	Nonbonding interactions—Van der Waals forces (≈−55 kcal/mol) and electrostatic interactions (≈−25 kcal/mol)—played a major role in mucoadhesion Chitosan and PAA demonstrated H‐bonding with the MUC molecule	Porosity‐controlled Multielemental transbuccal system	35
Poly(acrylic acid) Hydroxypropyl cellulose	Rotation of saccharide and acrylate residues producing strain due to steric interactions H‐bonds formed between the polymer matrix and the MUC	Ultrafast disintegrating wafer matrix	36

## 7 CONCLUSION

The above discussion and cited literature proved that not‐so‐complex and time‐efficient molecular mechanics simulations can provide an in depth account of the bonding and nonbonding interactions occurring within a biomedical device or system fabricated using biopolymers. The static‐lattice atomistic simulations and MMER also confirmed that there is a direct relationship between the in silico findings and the in vitro and/or in vivo results and hence atomistic simulations can be employed for the construction of an “in vitro—in vivo—ex vivo—in cyto—in silico” performance‐correlation profile within biomedical material assemblies.

## CONFLICT OF INTERESTS

The authors declare that they have no conflicts of interest with the contents of this article.
